# Quantitative trait loci associated with apple endophytes during pathogen infection

**DOI:** 10.3389/fpls.2023.1054914

**Published:** 2023-03-28

**Authors:** Amanda Karlström, Matevz Papp-Rupar, Tom A. J. Passey, Greg Deakin, Xiangming Xu

**Affiliations:** NIAB East Malling, East Malling, United Kingdom

**Keywords:** apple, microbiome, *Neonectria ditissima*, European canker, phyllosphere, *Malus x domestica*

## Abstract

The plant phyllosphere is colonized by microbial communities that can influence the fitness and growth of their host, including the host’s resilience to plant pathogens.There are multiple factors involved in shaping the assemblages of bacterial and fungal endophytes within the phyllosphere, including host genetics and environment. In this work, the role of host genetics in plant-microbiome assembly was studied in a full-sibling family of apple (Malus x domestica) trees infected with the fungal pathogen Neonectria ditissima. A Quantitative Trait Loci (QTL) analysis showed that there are multiple loci which influence the abundance of individual endophytic taxa, with the majority of QTL having a moderate to large effect (20-40%) on endophyte abundance. QTL regions on LG 1, 3, 4, 5, 10, 12, 13, 14 and 15 were shown to affect multiple taxa. Only a small proportion of the variation in overall taxonomic composition was affected by host genotype, with significant QTL hits for principal components explaining <8% and <7.4% of the total variance in bacterial and fungal composition, respectively. Four of the identified QTL colocalised with previously identified regions associated with tolerance to Neonectria ditissima. These results suggest that there is a genetic basis shaping apple endophyte composition and that microbe-host associations in apple could be tailored through breeding.

## Introduction

*Neonectria ditissima* is a fungal pathogen that causes wood cankers in apple and other broad-leaved trees (Weber, 2014; Saville et al., 2019) in most of the temperate areas of the world. A most damaging aspect of this disease is that latent infection established in nurseries can develop into main stem cankers post-planting, rapidly girdling the trees resulting in high tree mortality in young high intensity orchards. Given the withdrawal of several effective fungicides (e.g. carbendazim and copper-based products), host resistance is thus an important component in managing canker disease. Many modern apple varieties are highly susceptible to *N. ditissima* (Gomez-Cortecero et al., 2016; Papp-Rupar et al., 2022). Since host resistance against *N. ditissima* is quantitative (Gomez-Cortecero et al., 2016; Bus et al., 2019), it may take a long time to breed apple cultivars with effective resistance against the pathogen. The effects of identified apple scion Quantitative Trait Loci (QTL) on disease resistance are low to moderate, with 4.3 to 19% of variance explained by a single QTL (Karlström et al., 2022).

Many bacterial and fungal endophytes can improve tolerance of host plants to abiotic and biotic stresses and enhance their growth (Khare et al., 2018; Omomowo and Babalola, 2019; Dini-Andreote, 2020). A number of apple endophytes at leaf scars, a major entry site of *N. ditissima*, are associated with host susceptibility to *N. ditissima* (Olivieri et al., 2021). Furthermore, specific endophytes from apple show *in-vitro* antagonistic effects against *N. ditissima* (Liu et al., 2020). Apple genotypes can significantly influence endophyte species richness and composition (Hirakue and Sugiyama, 2018; Liu et al., 2018; Liu et al., 2020) and cultivars with a higher degree of relatedness share similarities in endophyte community (Liu et al., 2018). In addition to cultivar, environment and apple tissue type can also considerably influence endophyte composition (Liu et al., 2020; Olivieri et al., 2021).

Apple endophyte composition could potentially be altered through specific agronomic practices or augmentation of specific endophyte strains with biocontrol ability to reduce susceptibility to*. N. ditissima*. In breeding programmes, specific (desirable) endophytes may be included as a selection criterion to breed cultivars with disease suppressive endophyte composition. To adopt this second approach, we need to assess the magnitude of the heritability of endophyte communities and to exploit genetic markers for specific endophytic components.

In a previous paper (Papp-Rupar et al., 2022), we used F_1_ progeny trees derived from a cross between two moderately canker tolerant cultivars in an experimental orchard to determine the variability and heritability of bacterial and fungal endophyte communities in apple leaf scars. We also estimated correlations of endophytes with canker development. The results showed that specific components of endophytes as well as individual fungal/bacterial taxa in leaf scars were partially controlled by host genotypes. The broad sense heritability for specific aspect of endophytic composition and individual bacterial/fungal groups ranged between 0.05 to 0.37.

This study further investigates the host-genetic factors associated with endophytes and uses the metabarcoding data set from our previous publication (Papp-Rupar et al., 2022) to identify genetic regions involved in shaping apple endophyte composition. Through this analysis we aim to understand the genetic architecture of the interaction between the apple host and the microbial communities contained within its wood. The genetic mapping can also provide tools, in the form of molecular markers, to tailor microbe-host associations through breeding. Traits (Principal Components [PC]) of endophytes or abundance of individual endophyte taxa) that showed significant genetic variability among F_1_ progeny trees in the previous study were included in the QTL analysis in this study.

## Materials and methods

### Experimental design, canker assessment and endophyte profiling

Experimental materials and methods including orchard layout, canker inoculation, disease monitoring, profiling of fungal and bacterial endophytes, bioinformatic processing of amplicon sequences, and estimation of broad-sense heritability of endophytes were fully described in our previous publication (Papp-Rupar et al., 2022). The methodology is briefly described below to aid the understanding of this study.

A biparental cross between ‘Aroma’ x ‘Golden Delicious’ cultivars consisting of 56 genotypes including the two parents and 54 F1 genotypes were grafted on M9 EMLA rootstock. Four replicate trees were grown in a randomised block design in an orchard at East Malling, Kent, UK. Five leaf scars were inoculated per tree (one per shoot) with *N. ditissima* Hg199 isolate. The length of canker lesions was measured at 5, 8 and 11 months post inoculation (mpi) and the average canker lesion size at every used in further analysis (Karlström et al., 2022). The trees were also assessed at 20 mpi for percent canopy area with healthy foliage (Healthy Tree Area, %HTA) and percent branches with canker (Cankered Branches, %CB).

The samples for endophyte analysis were taken by dissecting 12 freshly exposed leaf scars per tree according to Olivieri et al. (2021). Sampled leaf scars were taken from four healthy, one-year-old shoots per tree. DNA was extracted (DNeasy Plant Mini kit, Qiagen) and subjected to amplicon sequencing of ITS1 (ITS1-1F/ITS2; White et al., 1990; Gardes and Bruns, 1993) and 16S V5-V7 (799F/1193R, Chelius and Triplett, 2001; Bodenhausen et al., 2013) regions.

Amplicon sequence data were processed to produce OTU representative sequences and frequency tables with the UPARSE pipeline (Version 10.0) (Edgar, 2013), as previously described (Papp-Rupar et al., 2022). The lowest taxonomic rank with a confidence of >= 80% has been used to describe the OTUs. The fungal and bacterial OTU abundancy tables were normalised by the median-of-ratios (MR) method implemented in DESeq2 (Love et al., 2014).

### Traits for QTL analysis

Variance in endophyte abundances attributed to the host genetic factor was statistically tested in the previous study (Papp-Rupar et al., 2022) by comparing a model with the genotypic component included with the model without (Chi square, df=1). Bacterial and fungal PCs and OTUs for which there was a significant host genotypic effect (α=0.05) found in previously published research (Papp-Rupar et al., 2022) were included in the QTL analysis in this study, namely, bacteria: 7 PCs and 47 OTUs, fungi: 4 PCs and 22 OTUs. Associations between traits were evaluated using the native R-function cor.test with Pearson correlation using data from individual trees. All statistical analyses were conducted in R (version 4.0.4; R Core Team, 2014). Average values across all four replicates were used for the QTL analysis.

Genotypic data for the apple biparental population and linkage map DNA from the biparental population and the two parental cultivars were extracted as per Karlström et al. (2022). The population was genotyped on the Illumina Infinium^®^ 20k SNP array. Genotype assignment was performed in GenomeStudio Genotyping Module 2.0 (Illumina). SNP filtering was conducted in ASSisT, leaving 11,000 SNPs to be used for further analysis.

### QTL analysis

The linkage map used for the analysis was produced in the OneMap package (Margarido et al., 2007). Markers were removed from the dataset if they had a distorted segregation, both parents were homozygous, or both parents had missing genotype data for the marker. To reduce the computational burden, markers with a pairwise recombination fraction of zero were collapsed into bins represented by the marker with the lower amount of missing data among those in the bin. A LOD score ≥ 3.0 and recombination fraction ≥ 0.50 were considered to indicate linkage between markers. Markers were ordered according to the iGL consensus linkage map (di Pierro et al., 2016) whereas genetic distances between markers were calculated by OneMap using the Kosambi mapping function. The relationship between segregations of single markers and traits were analysed with a Kruskal-Wallis test (K-W) using the ‘kruskal.test’ function and the Benjamini-Hochberg method (Benjamini and Hochberg, 1995) was used to adjust p-values for false discovery rate. QTL positions of significant K-W markers were determined as their position on the linkage map produced in Onemap. The position of markers which had been removed in the binning process were given by a marker from within the same bin. The percentages of phenotypic variation (R2) explained by QTLs were estimated in FullsibQTL. All significant QTL positions were included in the calculation of QTL effects for each phenotype.

Verification of the QTL mapping was performed through Composite Interval Mapping (CIM) in the FullsibQTL package as described previously (Gazaffi et al., 2014). A maximum of 10 cofactors were stipulated to locate QTLs. A random permutation test (α = 0.05; n = 1000 replicates) in FullsibQTL was used to determine the critical Logarithm of Odds (LOD) score for declaring the presence of a true QTL.

## Results

### Trait data

The studied endophyte abundance traits showed normal distributions in the studied population. Distributions of trait data for each trait associated with a QTL is shown in **Supplementary Figure 1**. Mean values for the biparental population and the two parents is shown in **Supplementary Table 2**, **3**. The correlation in the abundance of endophyte and canker disease traits are shown in **Supplementary Figure 2** to indicate the degree of correlation among these phenotypic traits. Further descriptions of trait data from the same experiment is detailed in Karlström et al. (2022) and Papp-Rupar et al. (2022) for disease and endophyte abundance results, respectively. Disease results for standard reference cultivars from the field experiment is detailed in Karlström et al. (2022).

### Linkage map

The linkage map had 8,032 SNP markers, which were divided into 3,681 bins to produce the map. Markers were distributed along 9,685 cM with an average distance between markers of 2.56 cM (**Supplementary Table 1**). The ordering of markers was forced to follow the map positions in the iGL consensus map produced by di Pierro et al. (2016). The linkage map from the Aroma x Golden Delicious population deviated substantially in size from the consensus map, as it was 8,418 cM longer and had a larger average distance between markers compared to the consensus map.

### QTL analysis

#### Bacterial endophytes

**Figure 1:**
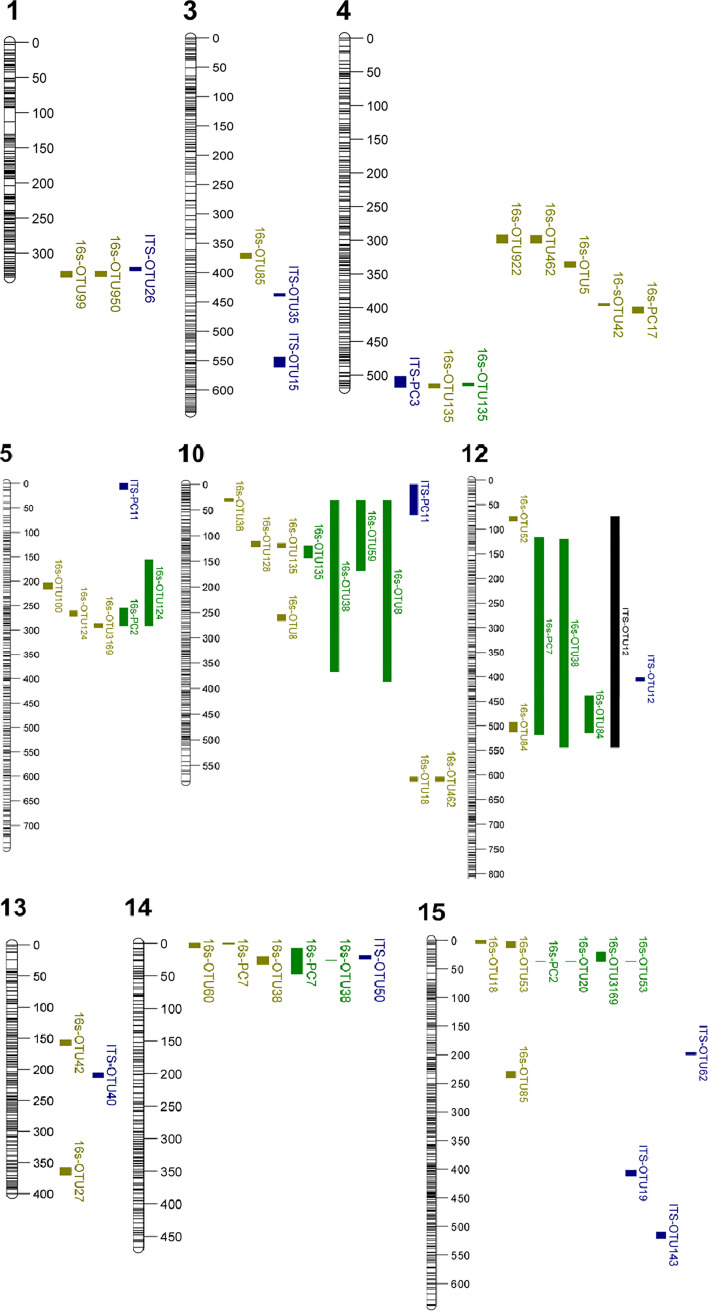
Linkage groups with QTL positions from the composite interval mapping (CIM) and Kruskal-Wallis (K-W) test. Phenotype colour indicates which analysis the QTL is associated with, yellow: 16s CIM analysis, blue: ITS CIM analysis, green: 16s K-W test, black: ITS K-W test. The figure only shows linkage groups with QTL interval supported by more than one trait.

There were significant QTLs associated with 21 of the 47 OTUs (**Tables 1**, **2**; **Supplementary Table 2**). These significant QTL hits were distributed over 15 linkage groups (LGs) with the QTL position on seven LGs (LG1, 4, 5, 10, 12, 14, 15) associated with multiple bacterial OTUs (**Figure 1**). The seven PCs representing specific bacterial endophyte communities in apple leaf scars were associated with QTL hits on five LGs, four of which were present at positions that were supported by multiple phenotypes. The CIM analysis resulted in 37 significant hits across all bacterial abundance traits and LGs (**Table 1**), whereas 17 significant marker-trait associations were identified with the K-W test (**Table 2**). The QTL positions on LG 4, 5, 10, 12, 14 and 15 were supported by both the CIM and K-W test. Only SNP marker alleles of QTL detected with both methods are described below.

**Table 1:** Quantitative trait loci (QTL) composite interval mapping for abundance data of bacterial (16S) and fungal (ITS) endophytes in apple leaf scar tissues of a full-siblings mapping population of a ‘Aroma’ x ‘Golden Delicious’ cross.

Trait	Taxonomy (> 80% confidence)	LG	Position (cM)		Maximum LOD	Maximum LOD-score	R^2^ (%)	Correlation to canker severity (%HTA)
			Start	Finish				
16S								
OTU644	Actinobacteria	16	439	442	442	11	1	
OTU27	Aurantimonadaceae	13	358	370	365	12	27	
OTU38	Aureimonas							0.22
		10	28	33	32	10	26	
		14	21	32	26	11	22	
OTU128	Bacteria	10	111	122	120	13	36	
OTU950	Bacteria							-0.095
		1	326	334	332	14	34	
		17	57	63	62	12	19	
OTU52	Comamonadaceae	2	102	114	110	14	36	
		7	129	132	74	10	12	
		12	74	84	81	10	27	
OTU18	Deinococcus	10	573	581	581	10	6	
		15	0	5	5	10	25	
OTU135	Deinococcus							0.07
		4	513	519	516	11	29	
		10	115	124	120	11	22	
OTU124	Deltaproteobacteria	5	261	271	267	14	31	
		8	505	508	508	10	6	
OTU5	Kineococcus	4	332	340	340	9	4	
OTU53	Marmoricola	15	2	13	7	11	37	
OTU462	Microbacteriaceae	4	293	304	299	12	27	
		10	573	581	579	11	6	
OTU42	Nocardioides	4	394	397	399	9	26	
		6	179	186	179	11	23	
		13	153	162	156	10	16	
OTU922	Proteobacteria	4	292	304	304	15	32	
OTU85	Pseudokineococcus	3	367	376	374	11	13	
		6	421	432	429	11	26	
		15	229	240	232	11	27	
OTU100	Rhodobacteraceae	5	203	216	213	12	29	0.11
OTU3169	Rhodospirillales	5	286	295	290	9	24	
OTU84	Roseomonas	12	493	512	499	13	34	0.10
OTU8	Sphingomonas	10	253	266	262	16	50	0.23
OTU99	Terracoccus	1	326	334	332	14	36	
		16	86	100	93	12	20	
OTU60	Williamsia	14	0	7	0	13	35	
PC16		8	63	75	71	13	35	
PC17		4	399	408	404	10	23	
PC7		14	0	1	0	10	36	0.26
ITS								
OTU143	Dothideomycetes	15	511	521	511	11	40	0.10
OTU35	Entyloma	3	436	440	438	11	44	-0.14
OTU50	Entyloma calendulae	14	19	25	25	12	35	-0.12
OTU12	Fungi	12	402	409	405	10	42	
OTU19	Fungi							-0.008
		6	531	546	536	13	27	
		15	402	412	402	12	15	
OTU40	Fungi	13	206	213	210	10	23	
OTU62	Phaeosphaeriaceae	15	197	200	199	10	3	
OTU26	Subplenodomus iridicola	1	321	324	322	13	19	0.003
OTU71	Taphrina							-0.05
		8	141	152	141	11	17	
		17	312	315	314	11	34	
OTU310	Taphrina	16	15	22	20	12	25	
OTU15	Tilletiopsis washingtonensis	3	544	561	549	11	30	
PC3								-0.19
		4	502	518	508	11	22	
		6	442	445	444	12	30	
PC11		5	0	13	4	10	7	
		10	0	59	9	17	20	

For traits with a significant correlation to canker severity, the Pearson correlation coefficient to % healthy tree area (%HTA) is shown.

**Figure 2:**
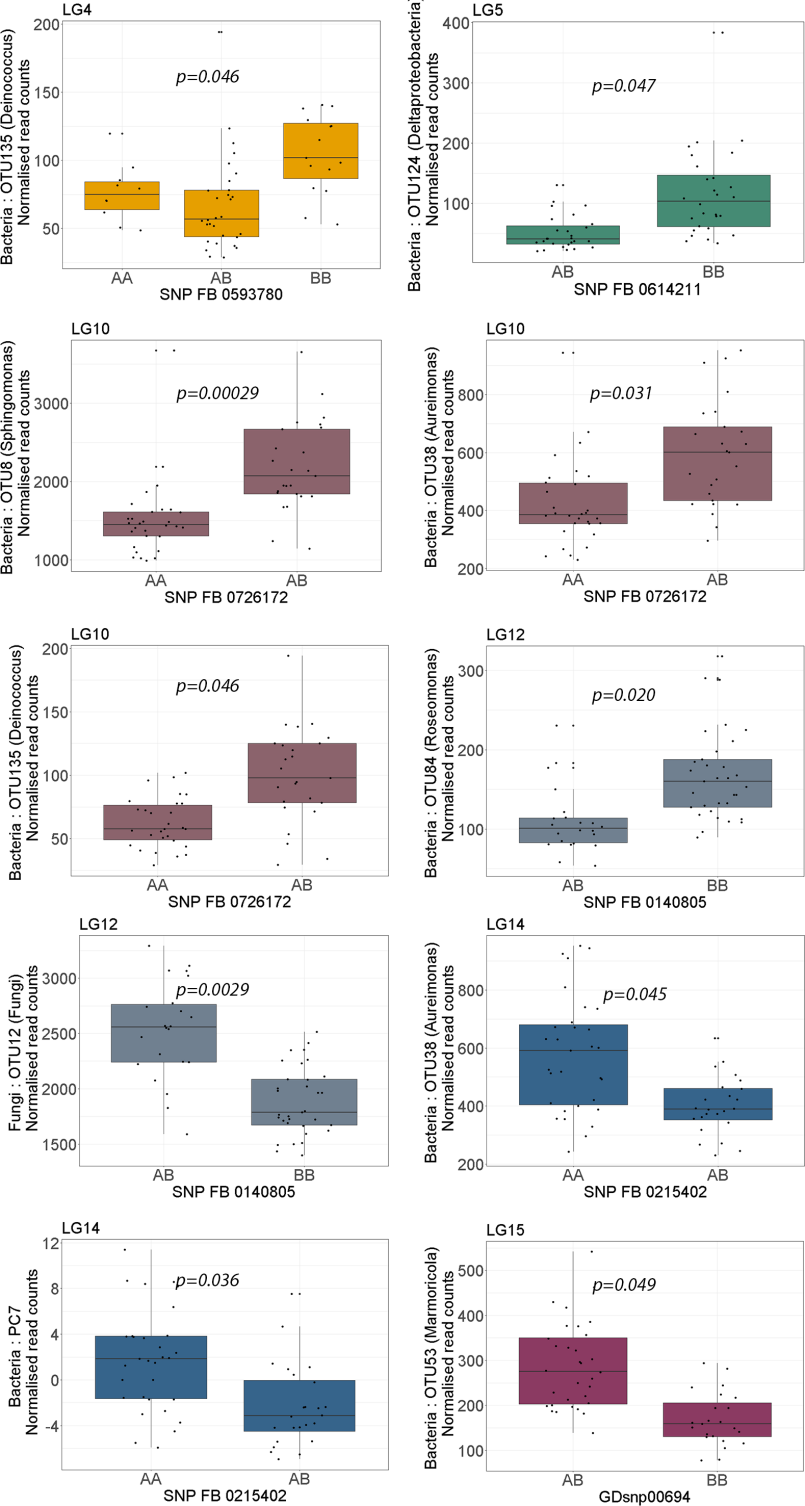
Boxplots showing the effect of phenotypic variation between SNP marker genotypes linked to QTLs for endophyte abundance. Kruskal-Wallis test was used to determine the significant differences between the mean values of SNP genotypes. Graph colour indicates the respective linkage group (LG) of the QTL.

A QTL on LG4 was associated with PC17 and the abundance of five bacterial OTUs: Proteobacteria (OTU922), Microbacteriaceae (OTU462), *Kineococcus* (OTU5), *Nocardioides* (OTU42) and *Deinococcus* (OTU135). The QTL position on LG4 of *Deinococcus* (OTU135) was supported by both the CIM and K-W test (**Tables 1**, **2**; **Figure 1**). A lower relative abundance of this OTU segregated with the ‘A’-allele of the SNP marker FB 0593780 (**Figure 2**), which was located in the centre of the QTL. The ‘A’-allele was inherited from both parents and seemed to have a dominant effect on the *Deinococcus* abundance.The QTL effect for LG4, as estimated from the CIM analysis, ranged from 4.5% to 32.0% (with an average of 23.0%) depending on bacterial abundance trait (**Table 1**).

**Table 2:** Quantitative trait loci (QTL) identified from Kruskal-Wallist test for abundance data of bacterial (16s) and fungal (ITS) endophytes in apple leaf scar tissues of a full-sibling mapping population of ‘Aroma’ x ‘Golden Delicious’ cross.

Trait	Taxonomy (> 80% confidence)	LG	Position (cM)		Correlation to canker severity (%HTA)
			Start	Finish	
16s					
OTU38	Aureimonas				0.22
		6	3	57	
		10	32	366	
		12	120	544	
		14	26	26	
OTU59	Bacteria	10	32	169	0.13
OTU135	Deinococcus				0.07
		4	512	516	
		10	120	143	
OTU124	Deltaproteobacteria	5	156	291	
OTU53	Marmoricola	15	37	37	
OTU3169	Rhodospirillales	15	20	37	
OTU84	Roseomonas	12	440	514	0.10
OTU20	Sphingomonadaceae	15	37	37	
OTU8	Sphingomonas	10	32	386	0.23
PC2		5	255	291	
		15	37	37	
PC7					0.26
		12	116	518	
		14	8	47	
ITS					
OTU12	Fungi	12	74	544	

For traits with a significant correlation to canker severity, the Pearson correlation coefficient to % healthy tree area (%HTA) is shown.

A QTL region on LG5 was associated with 16s PC2 and three OTUs: Rhodobacteraceae (OTU100), Deltaproteobacteria (OTU124) and Rhodospirillales (OTU3169) (**Figure 1**). The QTL position for Deltaproteobacteria OTU124 was supported by both the CIM and K-W test (**Tables 1**, **2**). The ‘A’-allele, inherited from ‘Golden Delicious’, for the SNP FB 0614211 was associated with a lower abundance of the Deltaproteobacteria OTU124 (**Figure 2**). The CIM analysis estimated the effect of the QTL on LG5 in the range of 24.0 to 31.0% (**Table 1**).

One region in the top of LG10 was associated with five OTUs: *Sphingomonas* (OTU8), *Aureimonas* (OTU38), *Deinococcus* (OTU135), and two OTUs (OTU59 and OTU128) that could only be assigned to the bacteria kingdom with 80% confidence (**Table 1**). The CIM QTL position was supported by K-W test for three OTUs – OTU8, OTU38 and OTU135 (**Table 2**; **Figure 1**). The SNP marker FB 0726172 had a significant effect on these three traits and the ‘B’-allele for this marker, inherited from ‘Aroma’, was associated with a higher abundance of the bacterial groups (**Figure 2**). A second QTL region, positioned at the bottom of LG10, was associated with the abundance of *Deinococcus* (OTU18) and Microbacteriaceae (OTU462). The effect of the QTL on LG10 was estimated to be in the range of 6.0% to 50.0%, with an average of 24.0% (**Table 1**).

Significant QTL hits were identified on LG12 for PC7 and three OTUs: *Aureimonas (*OTU38), Comamonadaceae (OTU52) and *Roseomonas* (OTU84). The QTL location on LG12 was supported by both the CIM and K-W test for *Roseomonas* OTU84 only. The ‘A’-allele of the significant SNP marker FB 0140805 was inherited from ‘Golden Delicious’ and associated with a lower abundance of *Roseomonas* OTU84. Interestingly, the same allele was linked to higher relative abundance of the fungal OTU12, for which taxonomic rank below kingdom could not be assigned (**Table 1**; **Figure 2**). The CIM estimated that the QTL on LG12 for OTU52 and OTU84 accounted for 27.0% and 34.0% of the total phenotypic variation, respectively (**Table 1**).

OTU60 (*Williamsia*), OTU38 (*Aureimonas*) and PC7 were associated with QTLs positioned on LG14. The QTL on LG14 was identified by both the CIM and K-W analysis for OTU38 and PC7 (**Tables 1**, **2**; **Figure 1**). The ‘A’-allele for the significant SNP marker FB 0215402 was, inherited from ‘Golden Delicious’ and linked with a lower abundance for both traits (**Figure 2**). The estimated QTL effects for LG14 ranged from 22.0% to 36.0% (**Table 1**).

A QTL position identified on LG15 was associated with PC2 and four OTUs: *Deinococcus* (OTU18), Sphingomonadaceae (OTU20), *Marmoricola* (OTU53) and Rhodospirillales (OTU3169). The QTL position for *Marmoricola* OTU53 was confirmed by both the CIM and K-W analysis (**Tables 1**, **2**; **Figure 1**). The SNP marker GDsnp00694 had a significant effect on the abundance of *Marmoricola* OTU53. The ‘A’ allele for this marker, which was inherited from ‘Golden Delicious’ was present in genotypes with a higher median abundance of *Marmoricola.* The QTLs on LG15 accounted for between 25.0% to 37.0% of the variation in bacterial OTUs and PCs (**Table 1**).

#### Fungal endophytes

There were significant QTLs associated with 13 out of the 22 fungal OTUs and four fungal PCs (**Tables 1**, **2**; **Supplementary Table 3**). QTLs associated with the abundance of fungal endophytes were identified on LG1, 3, 4, 5, 6, 8, 10, 12, 14, 15, 16 and 17. The majority of QTL hits were identified by the CIM analysis, whereas only one significant QTL location on LG12 (for OTU12 – Fungi) was identified by the K-W test.

A significant QTL associated with OTU26 (*Subplenodomus iridicola*) was identified on LG1. The same QTL position was also associated with two bacterial OTUs (OTU950 and OTU99, **Figure 1**). The QTL on LG1 explained 19% of the variation in abundance of *Subplenodomus iridicola* OTU26. Two QTLs were identified on LG3 for OTU15 (*Tilletiopsis washingtonensis*) and OTU35 (*Entyloma*), with the respective estimated QTL effect of 30.0% and 44.0% (**Table 1**).

The QTL location on LG12 associated with OTU12 (Fungi) was identified with both the CIM and K-W analysis and had a effect size of 42% (**Table 1**). A single fungal OTU had a QTL hit on LG13 (OTU40, Fungi), this LG was also associated with the bacterial endophyte traits OTU42 (Nocardioides) and OTU27 (Aurantimonadaceae). However, different genetic positions were identified for each of the three QTL (**Figure 1**).

Three fungal OTUs had significant QTL hits on LG15, with the estimated effects in the range of 3.0% to 40.0%. These fungal OTUs associated with LG15 were OTU19 (Fungi), OTU62(Phaeosphaeriaceae) and OTU143 (Dothideomycetes). The fungal endophyte QTL on LG15 do not overlap with the QTL associated with the bacterial endophyte traits.

Fungal PC3 had two QTL hits, one on LG4 which overlapped with QTL for the bacterial OTU135 (*Deinococcus*) and one on LG6. The QTL hits had estimated effect sizes of 22 and 30%, respectively (**Table 1**), and together account for over half of the genetic variation in this trait.

#### Canker phenotypes

There was no significant QTL associated with canker traits (lesion size, %HTA or %CB) from either CIM or K-W analysis.

### Association between QTL traits and severity of European canker

Significant correlations between endophyte abundance traits and European canker disease based on adjusted P-values were given in Papp-Rupar et al. (2022). Here we focused on the correlation between canker development and those microbial traits with significant QTLs identified. In addition, significance of correlations in this study was not adjusted as in the previous research. There was a significant correlation between OTU abundance/PC score and canker disease phenotypes for 11 of the 27 bacterial traits for which significant QTL were identified (**Tables 1** and **2**). The bacterial endophytes belonging to OTU59 (Bacteria), OTU38 (*Aureimonas*), OTU135 (*Deinococcu*s) and OTU8 (*Sphingomonas*) for which QTL regions positioned in the top of LG 10 were identified were positively correlated with %HTA (**Tables 1**, **2**). i.e. higher abundance was present in the trees with higher % of healthy canopy.). Similarly, bacterial endophyte traits that were associated with the QTL regions on LG 12 (*Roseomonas*-OTU84, *Aureimonas*-OTU38) and LG 14 (*Aureimonas*-OTU38, 16s-PC7) were positively correlated with %HTA (**Tables 1**, **2**).

## Discussion

A number of studies in maize, Arabidopsis, rye grass and lettuce have investigated the genetic control of endophytic community by the plant host (Horton et al., 2014; Faville et al., 2015; Wallace et al., 2018; Damerum et al., 2021). It has further been shown that specific genetic regions control the association between plants and beneficial microbes (Vidotti et al., 2019; Yassue et al., 2021; Brachi et al., 2022), and that there is variation in the phyllosphere microbiome between resistant and susceptible genotypes(Balint-Kurti et al., 2010; Ginnan et al., 2020; Xueliang et al., 2020). This is, however, the first study to investigate QTLs that potentially affect endophyte recruitment in woody tissues and their association with resistance to European canker.

### QTL associated with bacterial and fungal endophytes

Previously we reported significant effect of apple host genotype on 20% of the 200 most abundant bacterial OTUs and 13% of the 100 most abundant fungal OTUs (Papp-Rupar et al., 2022). The current study found that the abundance data of approximately 40-60% of these OTUs could be associated with QTLs in the host. The failure to detect QTLs for a proportion of the OTUs influenced by host genotype could be due to the presence of small effect QTLs for which the size of the studied population was not sufficient to identify, or due to a lack of markers segregating with the causative variation in the genetic region of interest.

The majority of identified QTLs had a moderate-large effect on OTU abundance with R^2^ -values in the range of 20-40%. This indicates the feasibility to breed cultivars with enhanced ability to harbour small number of specific endophytic taxa. The results from this study would need to be reproduced in additional sites and years as spatial and temporal variability is known to affect endophyte composition. It has been demonstrated that there is a large spatial variability in endophyte composition in above-ground plant tissues. For example, block within orchard and orchard site have significant effect on apple leaf scar community of the same apple genotypes (Olivieri et al., 2021). Differences in microclimatic conditions, management practice and the environmental inoculum (load and diversity) could be explain the observed differences. Site-specific soil factors, including pH, carbon content, and C:N ratio, were also found to affect endophyte community (Pacifico et al., 2019). Additionally, our unpublished data on apple leaf scar microbiomes sampled in spring and autumn suggests that both size and diversity of leaf scar communities change with season. Tree age has also been found to be an important factor affecting foliar endophytes (Yu et al., 2021). Taken together, the significance and effect size of QTLs identified here could be affected by the properties of local climatic conditions, soil properties, inoculum availability and tree age.

*Sphingomonas* (OTU8) was associated with a QTL on LG10 and positively correlated with %HTA in the present data, indicting a potential role in suppressing disease development or improving general plant health. We note that OTU8 abundance significantly correlated with canker traits in this study where a subset of 45 traits (with QTLs identified) were analysed, but not in the previous research that used 300 most abundant traits (Papp-Rupar et al., 2022). Higher number of traits analysed in the previous research necessitated correction for multiple tests – thus a higher correlation is needed to achieve statistical significance. A different *Sphingomonas* OTU (OTU72) was found to corelate with %HTA in the previous research, however, no QTL was found associated with it in this study. Moreover, we recently showed that several abundant *Sphingomonas* OTUs had an overall higher relative abundance in several *N. ditissima* resistant apple cultivars than in susceptible cultivars when assessed three times over two growing seasons at two sites (Xu, unpublished). Specific *Sphingomonas* strains are known to promote plant growth (Pan et al., 2016; Luo et al., 2019). Furthermore, one seed-endophytic strain, *Sphingomonas melonis* ZJ26, is naturally enriched in specific rice cultivars and confers resistance against a bacterial pathogen (Matsumoto et al., 2021).

The relative abundance of *Sphingomonas* OTU8 was associated with a large effect QTL (R^2 =^ 50%) and hence amenable to conventional breeding. The ‘B’ allele of the SNP marker FB 0726172, which is located within this QTL, was associated with a higher abundance of *Sphingomonas.* Thus, marker assisted breeding could be a feasible approach for manipulating the association of apple trees with this taxa, although the effect of this molecular marker would need to be confirmed in a wider range of germplasm.

*Aureimonas* (OTU38) was linked to QTL hits on LG10 and LG14, and positively correlated to tree health in plants inoculated with canker (%HTA). This genus has previously been found in increased abundance in the leaf phyllosphere of ash trees tolerant to the fungal disease ash dieback, *Hymenoscyphus fraxineus* (Ulrich et al., 2020). The inoculation of ash seedlings with *Aureimonas altamirensis* C2P003 had positive effect on the plant health and reduced ash dieback symptoms (Becker et al., 2022). Furthermore, a study has shown an isolate of *Aureimonas* to have antifungal activity towards rice blast, *Magnaporthe oryzae.* The two QTL identified in this study together accounted for 48% of the genetic variation of OTU abundance. Interestingly, the SNP allele inherited from ‘Aroma’ for FB 0726172 on LG10 was associated with a higher abundance of OTUs from *Sphingomonas*, *Aureimonas* as well as *Deinococcus.* The number of normalised read counts of these three bacterial groups were approximately double in ‘Aroma’ compared to ‘Golden Delicious’. Hence, this locus may have a role in promoting associations with multiple beneficial microbes and is of interest for further studies. *Sphingomonas* spp. and *Aureimonas* spp. would be good candidates to further explore in terms of effect on canker disease development as well as understanding the molecular mechanisms underlying the QTL identified in this study.

Only minor PCs were shown to be significantly affected by host genotype (Papp-Rupar et al., 2022). The variation in endophyte abundance attributed to PCs with significant QTL hits was <8% for bacterial and <7.4% for fungal PCs. Hence breeding may have limited scope to target changes in larger endophytic features/communities. This contrasts with studies of rhizosphere endophyte communities, where major PCs have been shown to be associated with host genotype (Deng et al., 2021; Oyserman et al., 2022). This could be due to a broader and more active interaction between roots and microbes (through root exudates and other mechanisms) compared to the very specific interaction of particular strains in above-ground tissues (e.g. leaf-scars).

The linkage map used in our study based on the ‘Aroma’ x ‘Golden Delicious’ population was highly inflated compared to the iGL consensus map from which the marker orders were derived (di Pierro et al., 2016). This is likely due to a higher number of co-segregating markers in the biparental population as there is a limited number of recombination events compared to the multiple populations used to produce the consensus map (N’Diaye et al., 2017)

### Co-localisation of endophyte QTL and genetic regions associated with susceptibility to European canker

Several QTL regions (on LG 5, 8, 15 and 16) associated with endophyte abundance and composition colocalize with previously identified QTLs for resistance to *N. ditissima* (Karlström et al., 2022). OTU3169 (*Rhodospirillales*) and the bacterial PC2 had two QTL hits colocalizing with canker QTLs (on LG5 and 15), whereas the other OTUs/PCs with overlapping hits were specific to one of the canker QTL regions. Only a subset of the endophyte QTL with significant correlation to canker traits had QTL hits that co-localised with previously reported resistance QTL (bacteria: OTU8, OTU38, OTU59, OTU100, OTU159, fungi: PC3, OTU19, OTU71, OTU143) However, the canker disease QTL reported by Karlström et al. (2022) had small to moderate effect sizes (4-19%). The effect sizes of QTL associated with the bacterial traits that were correlated with canker traits were comparably larger. It is therefore plausible that the population size used by Karlström et al. (2022) was not sufficient to identify some small effect QTL associated with canker disease traits, however, the biparental population used in this study was enough to detect endophyte QTL which had relatively larger effect sizes.

The mode of action of QTLs associated with endophyte abundance and *N. ditissima* resistance are so far unknown. Plant host factors that have been shown to affect the phyllosphere microbiome include plant immunity, signalling, cuticle formation and secondary metabolites (Kniskern et al., 2007; Vogel et al., 2016; Chen et al., 2020; Jacoby et al., 2021). Similar molecular functions may influence the abundance of specific endophytes as well as the ability to reduce *N. ditissima* spread within the plant. Indeed, genes that are differentially regulated in apple trees after *Neonectria* infection are involved in plant defences and cell wall modifications (Ghasemkhani, 2015). Further studies will be needed to fully decipher whether identified QTLs have a direct effect in shaping phyllosphere community, or whether the shift in microbial composition is due to microbe-*Neonectria* interactions or altered plant responses.

## Data availability statement

The sequence data presented in the study are deposited in the European Nucleotide Archive (ENA), accession number: PRJEB49633. The SNP data presented in the study are deposited European Variation Archive, accession number PRJEB54689. The linkage map, trait data for each genotype and SNP data can be found in a supplementary data file.

## Author contributions

XX conceived the study. XX, AK, TP and MP-R designed, planned and carried out the experiment. GD performed OTU clustering and taxonomy assignment. AK produced the linkage map and carried out the QTL analysis. AK and XX wrote the manuscript. All authors discussed the results and commented on the manuscript. All authors contributed to the article and approved the submitted version.

## Supplementary material

Supplementary Figure 1 Histograms of trait distributions in the biparental population.

Supplementary Figure 2 Correlation matrix of Pearson’s correlation coefficient between bacterial (in grey) and fungal (black) abundance traits, and severity of canker disease (in yellow). This data originates from a previous publication by Papp-Rupar et al. (2022).

## References

[B1] Balint-Kurti P. Simmons S. J. Blum J. E. Ballaré C. L. Stapleton A. E. (2010). Maize leaf epiphytic bacteria diversity patterns are genetically correlated with resistance to fungal pathogen infection. Mol. Plant-Microbe Interact. 23 (4), 473–484. doi: 10.1094/MPMI-23-4-0473 20192834

[B2] Becker R. Ulrich K. Behrendt U. Schneck V. Ulrich A. (2022). Genomic characterization of aureimonas altamirensis C2P003–a specific member of the microbiome of fraxinus excelsior trees tolerant to ash dieback. Plants 11, 3487. doi: 10.3390/plants11243487 36559599 PMC9781493

[B3] Benjamini Y. Hochberg Y. (1995). Controlling the false discovery rate: a practical and powerful approach to multiple testing. J. Roy Stat. Soc. B. 57, 289–300. doi: 10.1111/j.2517-6161.1995.tb02031.x

[B4] Bodenhausen N. Horton M. W. Bergelson J. (2013). Bacterial communities associated with the leaves and the roots of arabidopsis thaliana. PloS One 8 (2), e56329. doi: 10.1371/journal.pone.0056329 23457551 PMC3574144

[B5] Brachi B. Filiault D. Whitehurst H. Darme P. Le Gars P. Le Mentec M.. (2022). Plant genetic effects on microbial hubs impact host fitness in repeated field trials. Proc. Natl. Acad. Sci. 119 (30), 2201285119. doi: 10.1073/pnas.2201285119 PMC9335298 35867817

[B6] Bus V. G. M. Scheper R. W. A. Walter M. Campbell R. E. Kitson B. Turner L.. (2019). Genetic mapping of the European canker (*Neonectria ditissima*) resistance locus *Rnd1* from *Malus* ‘Robusta 5.’. Tree Genet. Genomes 15, 1–13. doi: 10.1007/s11295-019-1332-y 30546292

[B7] Chelius M. K. Triplett E. W. (2001). The diversity of archaea and bacteria in association with the roots of zea mays l. Microbial Ecol. 41, 252–263. doi: 10.1007/s002480000087 11391463

[B8] Chen T. Nomura K. Wang X. Sohrabi R. Xu J. Yao L.. (2020). A plant genetic network for preventing dysbiosis in the phyllosphere. Nature 580 (7805), 653–657. doi: 10.1038/s41586-020-2185-0 32350464 PMC7197412

[B9] Damerum A. Arnold E. C. Bernard V. Smith H. K. Taylor G. (2021). Good and bad lettuce leaf microbes? Unravelling the genetic architecture of the microbiome to inform plant breeding for enhanced food safety and reduced food waste. bioRxiv. doi: 10.1101/2021.08.06.455490

[B10] Deng S. Caddell D. F. Xu G. Dahlen L. Washington L. Yang J.. (2021). Genome wide association study reveals plant loci controlling heritability of the rhizosphere microbiome. ISME J. 15 (11), 3181–3194. doi: 10.1038/s41396-021-00993-z 33980999 PMC8528814

[B11] Dini-Andreote F. (2020). Endophytes: The second layer of plant defense. Trends Plant Sci. 25, 319–322. doi: 10.1016/j.tplants.2020.01.007 32191867

[B12] di Pierro E. A. Gianfranceschi L. di Guardo M. Koehorst-Van Putten H. J. Kruisselbrink J. W. Longhi S.. (2016). A high-density, multi-parental SNP genetic map on apple validates a new mapping approach for outcrossing species. HorticulT Res. 3, 1–13. doi: 10.1038/hortres.2016.57 PMC5120355 27917289

[B13] Edgar R. C. (2013). UPARSE: highly accurate OTU sequences from microbial amplicon reads. Nat. Methods 10, 996–998. doi: 10.1038/nmeth.2604 23955772

[B14] Faville M. J. Briggs L. Cao M. Koulman A. Jahufer M. Z. Koolaard J.. (2015). QTL analysis of host plant effects on fungal endophyte biomass and alkaloid expression in perennial ryegrass. Mol. Breed. 35 (8), 161. doi: 10.1007/s11032-015-0350-1 26203296 PMC4506467

[B15] Gardes M. Bruns T. D. (1993). ITS primers with enhanced specificity for basidiomycetes-application to the identification of mycorrhizae and rusts. Mol. Ecol. 2 (2), 113–118. doi: 10.1111/j.1365-294X.1993.tb00005.x 8180733

[B16] Gazaffi R. Margarido G. R. A. Pastina M. M. Mollinari M. Garcia A. A. F. (2014). A model for quantitative trait loci mapping, linkage phase, and segregation pattern estimation for a full-sib progeny. Tree Genet. Genomes 10, 791–801. doi: 10.1007/s11295-013-0664-2

[B17] Ghasemkhani M. (2015). Resistance against fruit tree canker in apple. doctoral thesis (Acta Universitatis Agriculturae Sueciae: Swedish University of Agricultural Sciences), 64. Available at: https://res.slu.se/id/publ/77464.

[B18] Ginnan N. A. Dang T. Bodaghi S. Ruegger P. M. McCollum G. England G.. (2020). Disease-induced microbial shifts in citrus indicate microbiome-derived responses to huanglongbing across the disease severity spectrum. Phytobiomes J. 4 (4), 375–387. doi: 10.1094/PBIOMES-04-20-0027-R

[B19] Gomez-Cortecero A. Saville R. J. Scheper R. W. A. Bowen J. K. de Medeiros H. A. Kingsnorth J.. (2016). Variation in host and pathogen in the *Neonectria/Malus* interactions; towards an understanding of the genetic basis of resistance to European canker. Front. Plant Sci. 7, 1365. doi: 10.3389/fpls.2016.01365 27695463 PMC5023678

[B20] Hirakue A. Sugiyama S. (2018). Relationship between foliar endophytes and apple cultivar disease resistance in an organic orchard. Biol. Control 127, 139–144. doi: 10.1016/j.biocontrol.2018.09.007

[B21] Horton M. W. Bodenhausen N. Beilsmith K. Meng D. Muegge B. D. Subramanian S.. (2014). Genome-wide association study of arabidopsis thaliana leaf microbial community. Nat. Commun. 5 (1), 1–7. doi: 10.1038/ncomms6320 PMC4232226 25382143

[B22] Jacoby R. P. Koprivova A. Kopriva S. (2021). Pinpointing secondary metabolites that shape the composition and function of the plant microbiome. J. Exp. Bot. 72 (1), 57–69. doi: 10.1093/jxb/eraa424 32995888 PMC7816845

[B23] Karlström A. Gómez-Cortecero A. Nellist C.F. Ordidge M. Dunwell J.M. Harrison R.J. (2022). Identification of novel genetic regions associated with resistance to European canker in apple. BMC Plant Biol. 22, 452. doi: 10.1186/s12870-022-03833-0 36131258 PMC9490996

[B24] Khare E. Mishra J. Arora N. K. (2018). Multifaceted interactions between endophytes and plant: Developments and prospects. Front. Microbiol. 9, 2732. doi: 10.3389/fmicb.2018.02732 30498482 PMC6249440

[B25] Kniskern J. M. Traw M. B. Bergelson J. (2007). Salicylic acid and jasmonic acid signaling defense pathways reduce natural bacterial diversity on arabidopsis thaliana. Mol. Plant-Microbe Interact. 20 (12), 1512–1522. doi: 10.1094/MPMI-20-12-1512 17990959

[B26] Liu J. Abdelfattah A. Norelli J. Burchard E. Schena L. Droby S.. (2018). Apple endophytic microbiota of different rootstock/scion combinations suggests a genotype-specific influence. Microbiome 6, 1–11. doi: 10.1186/s40168-018-0403-x 29374490 PMC5787276

[B27] Liu J. Ridgway H. J. Jones E. E. (2020). Apple endophyte community is shaped by tissue type, cultivar and site and has members with biocontrol potential against *Neonectria ditissima* . J. Appl. Microbiol. 128, 1735–1753. doi: 10.1111/jam.14587 31981438

[B28] Love M. I. Huber W. Anders S. (2014). Moderated estimation of fold change and dispersion for RNA-seq data with DESeq2. Genome Biol. 15, 550. doi: 10.1186/s13059-014-0550-8 25516281 PMC4302049

[B29] Luo Y. Wang F. Huang Y. Zhou M. Gao J. Yan T.. (2019). Sphingomonas sp. Cra20 increases plant growth rate and alters rhizosphere microbial community structure of arabidopsis thaliana under drought stress. Front. Microbiol. 10, 1221. doi: 10.3389/fmicb.2019.01221 31231328 PMC6560172

[B30] Margarido G. R. A. Souza A. P. Garcia A. A. F. (2007). OneMap: software for genetic mapping in outcrossing species. Hereditas 144, 78–79. doi: 10.1111/j.2007.0018-0661.02000.x 17663699

[B31] Matsumoto H. Fan X. Wang Y. Kusstatscher P. Duan J. Wu S.. (2021). Bacterial seed endophyte shapes disease resistance in rice. Nat. Plants 7, 60–72. doi: 10.1038/s41477-020-00826-5 33398157

[B32] N’Diaye A. Haile J. K. Fowler D. B. Ammar K. Pozniak C. J. (2017). Effect of co-segregating markers on high-density genetic maps and prediction of map expansion using machine learning algorithms. Front. Plant Sci. 8, 1434. doi: 10.3389/fpls.2017.01434 28878789 PMC5572363

[B33] Olivieri L. Saville R. J. Gange A. C. Xu X. (2021). Apple endophyte community in relation to location, scion and rootstock genotypes and susceptibility to European canker. FEMS Microbiol. Ecol. 97, 131. doi: 10.1093/femsec/fiab131 PMC8497447 34601593

[B34] Omomowo O. I. Babalola O. O. (2019). Bacterial and fungal endophytes: Tiny giants with immense beneficial potential for plant growth and sustainable agricultural productivity. Microorganisms 7, 481. doi: 10.3390/microorganisms7110481 31652843 PMC6921065

[B35] Oyserman B. O. Flores S. S. Griffioen T. Pan X. van der Wijk E. Pronk E.. (2022). Disentangling the genetic basis of rhizosphere microbiome assembly in tomato. Nat. Commun. 13, 3228. doi: 10.1038/s41467-022-30849-9 35710629 PMC9203511

[B36] Pacifico D. Squartini A. Crucitti D. Barizza E. Lo Schiavo F. Muresu R.. (2019). The role of the endophytic microbiome in the grapevine response to environmental triggers. Front. Plant Sci. 10, 1256. doi: 10.3389/fpls.2019.01256 31649712 PMC6794716

[B37] Pan F. Meng Q. Wang Q. Luo S. Chen B. Khan K. Y.. (2016). Endophytic bacterium sphingomonas SaMR12 promotes cadmium accumulation by increasing glutathione biosynthesis in *Sedum alfredii* hance. Chemosphere 154, 358–366. doi: 10.1016/j.chemosphere.2016.03.120 27065458

[B38] Papp-Rupar M. Karlstrom A. Passey T. Deakin G. Xu X. (2022). The influence of host genotypes on the endophytes in the leaf scar tissues of apple trees and correlation of the endophytes with apple canker (*Neonectria ditissima*) development. Phytobiomes 6 (2), 127. doi: 10.1094/PBIOMES-10-21-0061-R

[B39] R Core Team (2014). R: A language and environment for statistical computing. (Vienna, Austria: R Foundation for Statistical Computing). Available at: http://www.R-project.org/.

[B40] Saville R. J. Olivieri L. Xu X. Fountain M. (2019). “Fungal diseases of fruit: apple cankers in Europe,” in Integrated management of diseases and insect pests of tree fruit (Cambridge: Burleigh Dodds Science Publishing), 59–83.

[B41] Ulrich K. Becker R. Behrendt U. Kube M. Ulrich A. (2020). A comparative analysis of ash leaf-colonizing bacterial communities identifies putative antagonists of hymenoscyphus fraxineus. Front. Microbiol. 11, 966. doi: 10.3389/fmicb.2020.00966 32547506 PMC7273808

[B42] Vidotti M. S. Lyra D. H. Morosini J. S. Granato Í.S.C. Quecine M. C. Azevedo J. L. D.. (2019). Additive and heterozygous (dis) advantage GWAS models reveal candidate genes involved in the genotypic variation of maize hybrids to azospirillum brasilense. PloS One 14 (9), 0222788. doi: 10.1371/journal.pone.0222788 PMC6752820 31536609

[B43] Vogel C. Bodenhausen N. Gruissem W. Vorholt J. A. (2016). The arabidopsis leaf transcriptome reveals distinct but also overlapping responses to colonization by phyllosphere commensals and pathogen infection with impact on plant health. New Phytol. 212 (1), 192–207. doi: 10.1111/nph.14036 27306148

[B44] Wallace J. G. Kremling K. A. Kovar L. L. Buckler E. S. (2018). Quantitative genetics of the maize leaf microbiome. Phytobiomes J. 2 (4), 208–224. doi: 10.1094/PBIOMES-02-18-0008-R

[B45] Weber R. W. S. W. S. (2014). Biology and control of the apple canker fungus *Neonectria ditissima* (syn. *N. galligena*) from a northwestern European perspective. Erwerbs-Obstbau. 56 (3), 95–107. doi: 10.1007/s10341-014-0210-x

[B46] White T. J. Bruns T. Lee S. J. W. T. Taylor J. (1990). Amplification and direct sequencing of fungal ribosomal RNA genes for phylogenetics. PCR protocols: guide to Methods Appl. 18 (1), 315–322. doi: 10.1016/B978-0-12-372180-8.50042-1

[B47] Xueliang T. Dan X. Tingting S. Songyu Z. Ying L. Diandong W. (2020). Plant resistance and leaf chemical characteristic jointly shape phyllosphere bacterial community. World J. Microbiol. Biotechnol. 36 (9), 1–12. doi: 10.1007/s11274-020-02908-0 32803493

[B48] Yassue R. M. Carvalho H. F. Gevartosky R. Sabadin F. Souza P. H. Bonatelli M. L.. (2021). On the genetic architecture in a public tropical maize panel of the symbiosis between corn and plant growth-promoting bacteria aiming to improve plant resilience. Mol. Breed. 41, 63. doi: 10.1007/s11032-021-01257-6 PMC10236062 37309313

[B49] Yu Z. Ding H. Shen K. Bu F. Newcombe G. Liu H.. (2021). Foliar endophytes in trees varying greatly in age. Eur. J. Plant Pathol. 160, 375–384. doi: 10.1007/s10658-021-02250-7

